# Efficacy of smartphone application-based multi-domain cognitive training in older adults without dementia

**DOI:** 10.3389/fnagi.2023.1250420

**Published:** 2023-11-23

**Authors:** Jinju Cho, Dayeong An, Eunhye Cho, Daeun Kim, Ingyu Choi, Jihyun Cha, JongKwan Choi, Duk L. Na, Hyemin Jang, Juhee Chin

**Affiliations:** ^1^Department of Neurology, Samsung Medical Center, Sungkyunkwan University School of Medicine, Seoul, Republic of Korea; ^2^BeauBrain Healthcare, Inc., Seoul, Republic of Korea; ^3^Department of Neurology, KyungHee University Medical Center, Seoul, Republic of Korea; ^4^Department of Psychology, Georgia Institute of Technology, Atlanta, GA, United States; ^5^OBELAB, Inc., Seoul, Republic of Korea; ^6^Department of Neurology, Seoul National University Hospital, Seoul, Republic of Korea

**Keywords:** computerized cognitive training, smartphone application-based multi-domain cognitive training, home training, functional near-infrared spectroscopy, prefrontal lobe activation, older adults

## Abstract

**Background:**

As the population ages and the prevalence of dementia increases, there is a growing emphasis on the importance of cognitive training to prevent dementia. A smartphone application-based cognitive training software program, BeauBrain Trainer (BBT), has been developed to provide better access to cognitive training for older adults. Numerous studies have revealed the effectiveness of cognitive training using a cognitive assessment tool. However, relatively few studies have evaluated brain activation using brain imaging as a result of improved cognitive function.

**Methods:**

All participants were required to download the BBT, an Android-based application for cognitive training, onto their own smartphone or tablet computer and to engage in cognitive training at home. Older adults without dementia were enrolled in this study, including 51 participants in the intervention group and 50 participants in the control group. The BBT comprised a set of 12 cognitive tasks, including two tasks in each of the following six cognitive domains: attention, language, calculation, visuospatial function, memory, and frontal/executive function. Each cognitive task was divided into four blocks based on its level of difficulty. A 16-week cognitive training was designed to carry out cognitive tasks using a total of 48 blocks (12 tasks × 4 levels) for at least 1.5 h per day, 5 days per week. All participants in the intervention group were given BBT tasks that gradually increased in difficulty level, which they submitted through a smartphone application daily for 16 weeks. The researchers monitored the participants’ task performance records on the website and encouraged participants to engage in cognitive training through regular contact. This study was conducted to investigate the improvement in cognitive function and the activation pattern of the frontal cortex in older adults participating in smartphone application-based cognitive training. The cognitive assessment tool was the BeauBrain cognitive screening test (CST), a tablet-based computerized cognitive screening test. The activation pattern of the frontal cortex was measured using functional near-infrared spectroscopy (fNIRS). Additionally, this study aimed to determine the positive effects of cognitive training on everyday functioning and psychological states using a questionnaire.

**Results:**

Of 101 participants, 85 older adults without dementia (84.1%) who completed the study protocol were included in the statistical analysis. There were 41 participants (80.3%) in the intervention group and 44 participants (88.0%) in the control group. A two-way repeated-measures analysis of variance (ANOVA) was used to compare the cognitive scores over a 16-week period between the intervention and control groups. According to the CST results, the intervention group exhibited a statistically significant increase in the language subtest scores, specifically the phonemic word fluency test, compared to those of the control group. The fNIRS results revealed greater activation in the dorsolateral prefrontal cortex during the STROOP incongruent task in the intervention group than did the control group. However, the effectiveness of cognitive training was not observed across a variety of rating scales, including everyday functioning, depression, self-efficacy, attention, and subjective memory complaints.

**Conclusion:**

This study revealed that a smartphone-based cognitive training application led to improvements in phonemic generative naming ability and activation of the prefrontal cortex in older adults without dementia. This study is meaningful because it confirmed that cognitive training is partially effective in enhancing frontal lobe function. It also provided information on the brain mechanisms related to the effects of cognitive training using fNIRS.

## 1 Introduction

The number of dementia patients continues to rise due to increasing life expectancy and the aging of the world’s population. According to the 2019 World Health Organization (WHO) guideline for reducing the risks of cognitive decline and dementia (World Health Organization, 2019), there are approximately 50 million people with dementia worldwide, and the dementia population is expected to increase to 82 million in 2030 and to 152 million in 2050. Korea has one of the fastest growing older adult populations, with the proportion of individuals aged ≥65 years accounting for 15.8% of the total population. The estimated number of people with dementia among the population ≥ 65 years of age is approximately 830,000, with a prevalence of 10.2%. The number of people with dementia in Korea is expected to continue to increase and to exceed 3 million by 2050. As a result, the total annual national dementia management cost is expected to increase from the current 17.3 trillion Won to 56.9 trillion Won, and the social burden increases daily (Lee et al., 2021).

Recent research indicates that enhancing educational access and implementing effective strategies to reduce the prevalence of vascular risk factors can potentially reduce the incidence of Alzheimer’s disease (Norton et al., 2014). It is anticipated that our society’s future will experience positive change through actions related to dementia prevention, intervention, and care (Livingston et al., 2017). The global policy to prevent dementia is an active approach to maintain and promote cognitive health, rather than a passive approach to manage dementia. The WHO guidelines recommend the following 12 methods for dementia prevention: physical activity, tobacco cessation, nutritional intervention, intervention for alcohol use disorders, social activity, cognitive interventions, weight management, management of hypertension, diabetes mellitus, dyslipidemia, depression, and hearing loss (World Health Organization, 2019). In particular, there is increasing interest in cognitive intervention for older adults at high risk of dementia. Cognitive intervention is divided into training, rehabilitation, and stimulation according to subject and content (Clare and Woods, 2003, 2004). Cognitive training is applicable to older adults with normal cognition, mild cognitive impairment, or dementia. Tasks of varying difficulty are performed depending on the degree of cognitive decline. Cognitive training provides structured tasks of attention, language, calculation, visuospatial functions, memory, and frontal/executive functions for varying abilities. This intervention is used to maintain and improve the abilities in specific cognitive domains that have been impaired. Cognitive rehabilitation is applicable to older adults with mild cognitive impairment or to those with mild to moderate dementia. This intervention is used to improve the activities of daily living (ADL) and to enhance performance and function in relation to collaboratively established behavioral or functional goals. Cognitive stimulation encompasses a wide range of interventions; its treatment to the programs are less standardized than are those of cognitive training and cognitive rehabilitation. This intervention is often applied to patients with moderate to severe dementia, rather than patients with mild cognitive impairment or those with early dementia. It is mainly used in a clinical, residential care, or daycare setting. The aim of cognitive stimulation is to improve orientation, activate cognition, and encourage participation. Cognitive stimulation includes various activities, such as word games, puzzles, music, gardening, and cooking. In this study, we selected cognitive training to maintain and improve the current cognitive function with the goal of maintaining and improving the damaged cognitive domain(s) in older adults with mild cognitive impairment and those with normal cognition. The study aimed to provide comprehensive cognitive training that encompassed various domains, including memory, attention, language, calculation, visuospatial functions, and frontal/executive functions.

Many studies have shown that cognitive training, which focuses on older adults with normal cognition or mild cognitive impairment dementia, is effective for improving cognitive function (Ball et al., 2002; Clare et al., 2010; Theill et al., 2013; Hill et al., 2017). Cognitive training methods are diverse and range from paper and pencil training to computerized cognitive training programs for digital devices such as robots, virtual reality, web-based, and mobile applications (Kim et al., 2015; Shah et al., 2017; Shellington et al., 2017; Klimova and Valis, 2018; Irazoki et al., 2020; Bonnechère et al., 2021; Zhong et al., 2021). Such methods have led to significant improvements in the cognitive abilities of older adults.

Currently, brain imaging technologies are most commonly used to determine brain lesions in neurodegenerative diseases or to assess the effects of cognitive training. In particular, functional brain imaging displays the brain activation patterns during cognitive tasks and provides pertinent information about brain mechanisms that are enhanced through cognitive training. This technology has been demonstrated to be a crucial tool for understanding the changes in neural mechanisms that underlie aging and Alzheimer’s disease (Belleville and Bherer, 2012).

Previous studies have provided evidence that brain activation is inversely proportional to symptoms in older adults and disease severity. Several studies have reported that patients with early-stage mild cognitive impairment (MCI) exhibited higher brain activation than did those with late-stage MCI (Celone et al., 2006; Clément et al., 2010; Clément and Belleville, 2010, 2012). Disease progression from MCI to Alzheimer’s disease has been reported to lead to lower brain activation. Decreased brain activation was detected in the medial temporal lobe (Sperling, 2007; Dickerson and Sperling, 2008; Pihlajamaki et al., 2009), as well as in some regions of the prefrontal cortex in patients with Alzheimer’s (Devous, 2002; Clément et al., 2010). Clément et al. (2010) described differences in cerebral activation patterns during episodic memory encoding and retrieval between 12 participants with MCI and 10 healthy participants. In the MCI group, increased activation in the left ventrolateral prefrontal cortex was observed. Decreased activation was observed in the brain areas that were either structurally compromised or hypometabolic in patients with Alzheimer’s disease. In a different study by Clément and Belleville (2010), memory-related activations during the verbal learning of semantically related or unrelated word pairs were investigated in 26 participants with MCI and 14 healthy participants. The MCI group was divided into two subgroups: MCI higher-cognition and MCI lower-cognition. The MCI higher-cognition subgroup showed increased activation in the right ventrolateral and dorsolateral prefrontal cortex than did the control group. In the MCI lower-cognition subgroup, no prefrontal activation was observed, and reduced activation in the posterior area was observed compared to that of the control group. A systematic review by Miotto et al. (2018) included several studies that evaluated the effects of cognitive training using fMRI in patients with amnestic single- or multiple-domain MCI. Three studies employed rehearsal-based strategies as the primary intervention, all of which were focused on computerized cognitive training. Four studies investigated neurophysiologic and cognitive changes associated with memory strategy training using fMRI. Most of the studies included in this systematic review revealed improvements in objective cognitive performance associated with cognitive training. Additionally, these studies exhibited increased activation in the temporal and prefrontal cortex associated with interventions in both typical and atypical brain areas and networks related to memory (Belleville et al., 2011; Balardin et al., 2015).

While there are numerous studies that have investigated the relationship of disease stage and neural mechanisms using brain imaging, relatively few have examined the brain mechanisms underlying the impacts of cognitive training. There is a variation in the interpretation of brain activation across studies. Functional MRI generates valuable data, but it offers poor temporal resolution of the BOLD signal and is very expensive. Therefore, using fMRI data as the outcome measure in large-scale randomized controlled trials poses a burden. Furthermore, the majority of cognitive training programs predominantly focuses on memory training that utilizes restorative and compensatory strategies. Moreover, if patients are unable to access institutional facilities for cognitive training, accessibility and usability are compromised. Cognitive training has been demonstrated in several studies to have a positive impact on enhancing the psychological well-being and quality of life of older adults (Giuli et al., 2016; Silva et al., 2021). However, there is limited evidence that cognitive training effectively improves everyday functioning (Ball et al., 2002; Kim and Lim, 2016). Extended follow-up observations are required to determine the effects of cognitive training on everyday functioning.

Therefore, the purpose of this study was to investigate the impact of a smartphone application based multi-domain cognitive training, conducted at home over 16 weeks, on the cognitive function and activation of the frontal lobe in older adults. Participants were able to perform cognitive training using this digital tool without any time or space constraints. Additionally, we utilized the relatively cost-effective functional near-infrared spectroscopy (fNIRS) to measure and analyze neural activity responses after cognitive training and identify brain activation patterns. Finally, we aimed to determine the effectiveness of cognitive training on improving older adults’ everyday functioning and psychological state.

## 2 Methods

### 2.1 Study design

This was a single-center, randomized, single-masked, and parallel-group study designed to investigate the effects of a smartphone application–based cognitive training tool in older adults without dementia. All participants were recruited from the Department of Neurology at Samsung Medical Center between July and October 2019. Eligible participants were randomly assigned to an intervention group and a control group. Randomization was stratified according to age (three age categories, 60–69, 70–79, and ≥80 years) and sex in a 1:1 ratio (Jang et al., 2021). Further details are provided in the Supplementary material.

There were six small subgroups within the intervention group according to the training starting point. The cognitive training program was conducted from October 2019 to March 2020 with 16 weeks of intervention with the BeauBrain Trainer (BBT) smartphone cognitive training application per group. The control group was provided BBT tasks for 16 weeks with the same contents as the intervention group after the post-test.

To investigate the effects of cognitive training, the primary and secondary outcome measures (see section 2.5, Outcome measures, for details) were conducted at baseline and 16 weeks later. All participants provided written consent to participate in the study before conducting the baseline test. The study protocol was approved by the Institutional Review Board (IRB) of Samsung Medical Center (IRB no. 2019-06-083-006).

### 2.2 BeauBrain trainer

The BBT is an Android-based cognitive training software program that is implemented as an application on a smartphone or tablet computer. The cognitive training tasks of the BBT have four difficulty levels (elementary, beginner, intermediate, and advanced) so that it can be provided to all older adults ranging from individuals with normal cognition to those with mild dementia. Two tasks were developed for each cognitive domain, and a total of 12 cognitive training tasks from the six cognitive domains of attention, language, calculation, visuospatial function, memory, and frontal/executive function was included in the BBT. The details of the BBT cognitive training tasks are presented in Table 1.

**Table 1:** Detailed explanation of BeauBrain Trainer’s 12 cognitive training tasks.

Cognitive domain	Task title	Description	Expected brain activation areas
Attention	Miracle of concentration	Count cards matching a target number and at the same time find the number of stimulus shapes within them. Stimuli come in a variety of shapes, patterns, and colors.	Frontal lobe
	Mental imagery of Korean letters	Count strokes (horizontal, vertical, diagonal, circle) in four-letter idioms, with increasing difficulty as letters disappear. Also, guess idioms based on their meanings.	Frontal lobe and right parietal lobes
Language	Word generation from initial consonants	Create words using two initial consonants from presented Korean characters. It consists of 144 combinations of initial consonants.	Left frontal lobes and temporal lobes
	Word-completion task	Guess words matching a given topic and initial consonant. There are a total of 157 topics.	Left frontal lobes and temporal lobes
Calculation	Calculation after cracking the code	Perform mental calculations by creating formulas using predetermined numbers for each symbol.	Frontal lobe and Left parietal lobe
	Arithmetic calculation	Mathematics calculation includes addition, subtraction, multiplication, division, and mixed.	Left parietal lobe
Visuospatial function	Block design	Stack blocks in 3D space to match the presented shapes, analyzing them from various perspectives (top, side, front).	Right parietal lobe
	Masterpiece jigsaw puzzle	Memorize features of famous paintings and the artist’s name, complete puzzle pieces, and guess the artist’s name within a limited time.	Right parietal lobe and Temporal lobe
Memory	Matching personal information	Remember various personal details, such as people’s faces, names, favorite foods, and favorite exercises.	Temporal lobe
	Find the same card	Remember and find the location of the same picture card within a limited time.	Temporal lobe
Frontal/executive functions	Coin combination exercise	Estimate the quantity of each coin needed to meet the conditions for a given number of coins and the total amount.	Frontal lobe and Left parietal lobe
	Weight inference	Deduce the weight needed on one side of the scale to balance the scales on both arms.	Frontal lobe

### 2.3 Participants

All participants were older adults without dementia recruited from the Department of Neurology, Samsung Medical Center. Older adults with no dementia included both those with normal cognition and those with mild cognitive impairment (MCI). The criteria for MCI were based on Petersen’s criteria (Petersen et al., 1999).

The following inclusion criteria were applied: (1) age ≥ 60 years; (2) literate with ≥6 years of education; (3) Korean version of Mini-mental State Examination (K-MMSE) score ≥ 24 points; (4) preserved activities of daily living (ADL), as defined by Seoul Instrumental ADL score < 8 points; and (5) Android smartphone user (as BBT is an Android-based application).

Meanwhile, participants with the following conditions were excluded from the study: (1) major cardiovascular events, such as stroke or myocardial infarction, in the past 3 months; (2) severe or unstable medical disease that could interfere with successful study completion; (3) severe hearing difficulty or visual disturbance; (4) limitations in communication; (5) previous dementia diagnosis; and (6) participation in another cognitive training session within 6 months of study enrollment.

### 2.4 Procedure

After installing the BBT application on their devices, participants in the intervention group had a one-week try-and-adapt period, during which they were trained on how to use and operate the BBT application. The intervention group performed cognitive training at home that was assigned to BBT through a mobile phone app for an average of 1 h 30 min daily, 5 days a week for 16 weeks. The participants were required to complete their training task before midnight every day. The BBT consisted of 12 tasks, two from each of the six cognitive domains. Each task has four difficulty levels of elementary, beginner, intermediate, and advanced. Forty-eight blocks (12 tasks × 4 levels) were used to create cognitive tasks for 16 weeks. Every day, there is a designated cognitive task, and all research participants follow a planned training schedule to conduct home training. During weeks 1–4, participants adapted to the 12 tasks at BBT in the elementary and beginner difficulty tasks. During weeks 5–12, they learned and applied the cognitive strategies within the intermediate and advanced difficulty levels. During weeks 13–16, high-level learning strategies were applied by increasing the level of difficulty to advanced.

The cognitive training tasks consisted of two sets (A and set B), each of which contained six tasks, including one from each cognitive domain. The intervention group performed elementary-and beginner-level tasks for the first 4 weeks and were required to complete 6–10 assigned tasks per day. Then, during weeks 5–8, the beginner-and intermediate-level tasks were completed (10–13 tasks per day). Intermediate-and advanced-level tasks were performed during weeks 9–12 (13–16 tasks per day), and advanced-level tasks were completed during weeks 13–16 (17–20 tasks per day). During weeks 1–12, cognitive tasks in sets A and B were assigned alternatively for 2 weeks. During the last 4 weeks (weeks 13–16), all cognitive tasks in both sets were performed (see Table 2 for details).

**Table 2:** Weekly/monthly cognitive task difficulty configuration.

Duration	Level	Cognitive task set	Number of training tasks
1–4 weeks	Elementary and beginner	1–2 weeks: Set A 3–4 weeks: Set B	6–10 tasks
5–8 weeks	Beginner and intermediate	5–6 weeks: Set A 7–8 weeks: Set B	10–13 tasks
9–12 weeks	Intermediate and advanced	9 weeks: Set A 10 weeks: Set B 11 weeks: Set A 12 weeks: Set B	13–16 tasks
13–16 weeks	Advanced	All sets	17–20 tasks

Set A: Word generation from initial consonants, mental imagery of Korean letters, matching personal information, block design, mental arithmetic calculation, and coin combination exercise. Set B: Word-completion task, miracle of concentration, identification of the same card, masterpiece jigsaw puzzle, calculation after cracking the code, and weight inference.

To facilitate adherence to this cognitive training, the daily task-completion rate was monitored every day. Participants who did not complete the assigned tasks were contacted through text messages and phone calls to encourage their engagement. Furthermore, the participants in the intervention group shared their experiences with the cognitive training and learned cognitive strategies from neuropsychologists by participating in offline sessions once per month. Participants were required to complete ≥80% of the assigned BBT tasks each day. Those who could not meet this requirement for >20% of the total training periods (16 of 80 days total) were excluded from the final study analysis.

The control group was not given any cognitive training task for 16 weeks. The primary and secondary outcome measures were performed at baseline and after 16 weeks.

### 2.5 Outcome measures

#### 2.5.1 BeauBrain cognitive screening test

Cognition was assessed by the BeauBrain Cognitive Screening Test (CST). The primary outcome measure was the total score of the BeauBrain CST, and the secondary outcome measures covered five cognitive domains of the BeauBrain CST: attention, language, visuospatial function, memory, and frontal/executive functions. The BeauBrain CST is a tablet-based computerized cognitive screening test consisting of seven neuropsychological subtests—the Visual Span Test (VST) forward and backward tasks which assesses attention; the Difficult Naming Test (DNT), semantic (fruits) and phonemic (Korean alphabet digeut) word fluency test for language; the Block Design Test for visuospatial function; time orientation and the Word Place Association Test (WPAT) for memory; and the Korean Trail-making Test—older adults Version (K-TMT-E) for frontal/executive function (Chin et al., 2020).

#### 2.5.2 Functional near-infrared spectroscopy

The activity of the frontal lobe was measured with fNIRS. The hemodynamic response of the prefrontal cortex was recorded during the cognitive tasks using a portable, wireless continuous-wave near-infrared spectroscopy system (fNIRS; OBELAB, Inc., Seoul, Korea; Shin et al., 2017; Kim et al., 2018). The device was composed of 24 sources (laser diodes) emitting two wavelengths (780 and 850 nm) and 32 photo-detectors in which a source and a detector in each pair were separated by 3 cm, forming 48 channels with a sampling rate of 8.138 Hz (Choi et al., 2016). The raw signals were first filtered by a band-pass filter (0.005–0.1 Hz) to minimize potential environmental artifacts and physiological noise from body movement. After the optical density of each wavelength was derived, the relative concentration changes of oxy-and deoxy-hemoglobin were calculated via the modified Beer–Lambert law (Delpy et al., 1988). Channels with poor signal quality [coefficient of variation <7.5%; low-frequency values <0.3 where the correlation between oxy-Hb and deoxy-Hb was < −0.9 (Takizawa et al., 2014)] were excluded from further analysis. The concentration values were block-averaged using values 5 s prior to each block as a baseline. Subsequent analyses were mainly conducted on oxy-Hb concentration changes. Participants performed the following three cognitive tasks: the Digit-Span Task (DST), Stroop Task (STROOP), and Social Event Memory Task (SEMT). Each task consisted of two blocks of 2 min separated by a 30-s rest period. For the statistical tests, block-averaged oxy-Hb changes from each channel were calculated to form eight regions of interest (ROIs) to yield more stable signals from each participant and to reduce the number of comparisons in subsequent tests. The locations of the fNIRS channels within the Brodmann area are presented in Supplementary Figures S1, S2.

### 2.6 Secondary outcome measures

The secondary outcome measures included various scales, as follows: the K-MMSE (Kang, 2006), the Korean Everyday Cognition (K-ECog; Farias et al., 2008; Song et al., 2019), the Bayer ADL (B-ADL; Hindmarch et al., 1998; Choi et al., 2003), the Subjective Memory Complaints Questionnaire (SMCQ; Schofield et al., 1997; Youn et al., 2009), the Korean version of the Geriatric Depression Scale (GDepS; Yesavage et al., 1982; Bae and Cho, 2004), the Korean version of the WHO Quality of Life Scale (Abbreviated Version; WHOQOL-BREF; Group W, 1998; Min et al., 2000), the Self-efficacy Scale (SES; Sherer et al., 1982), and the Attention Questionnaire Scale (AQS; Kim et al., 2011).

### 2.7 Statistical analysis

To analyze differences in demographic characteristics and S-IADL scores between the intervention and control groups, the chi-square test and independent *t*-test were used. The primary and secondary outcome measures of the BeauBrain CST and the fNIRS were analyzed using two-way repeated-measures analysis of variance (ANOVA) to evaluate differences between the two groups (the intervention and control groups) at two time points (baseline and after 16 weeks of cognitive training). Due to unintended technical issues in fNIRS analysis, data from 15 participants (DST: 3 trials, SEMT: 9 trials, STROOP: 3 trials) were excluded, and the data from 72 participants were included in the results. There were 40 participants in the intervention group and 32 in the control group. The resulting eight ROIs were the right and left dorsolateral prefrontal cortices (DLPFCs), right and left frontopolar cortices (FPCs), right and left orbitofrontal cortices (OFCs), and right and left ventrolateral prefrontal cortices (VLPFCs). Because most of the channels (channels with a 40–50% rejection rate) in the bilateral VLPFCs were rejected during preprocessing, the formal statistical tests were performed on regions other than the bilateral VLPFC. The mean signals from the left and the right hemispheres and overall prefrontal cortex were also examined. The fNIRS statistical results were analyzed excluding outliers using a 1.5 Inter Quartile Range (IQR) criterion. The criterion for statistical significance was *p* < 0.05. All statistical analyses were performed using R statistical software (R Core Team, 2013).

## 3 Results

### 3.1 Participants in the final analysis

**Figure 1:**
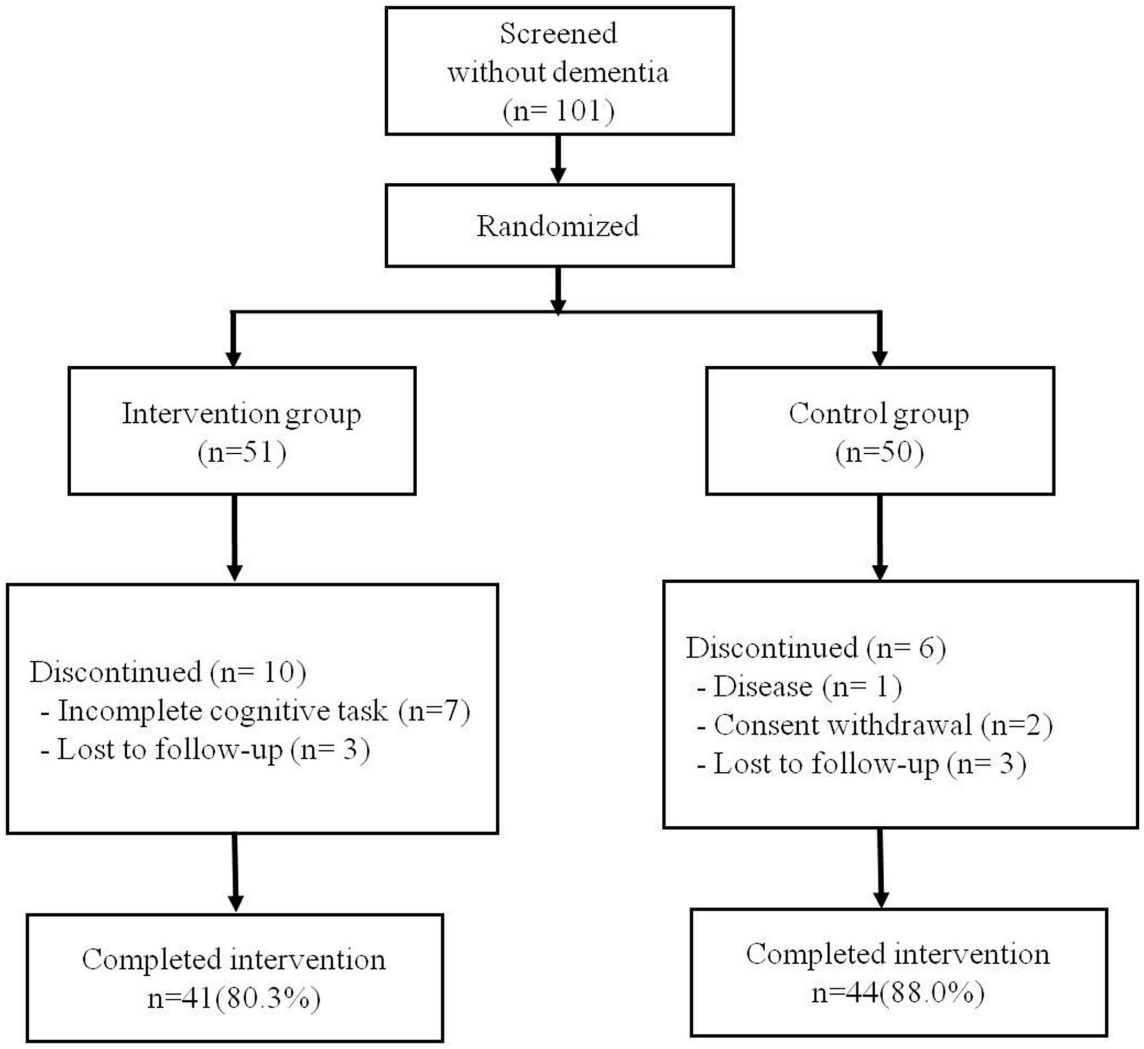
Enrollment and randomization.

A total of 101 older adults without dementia was randomly assigned, including 51 in the intervention group and 50 in the control group. Of the 51 intervention participants, seven (13.7%) were excluded because their rate of cognitive training completion was <80%. Three additional participants (5.8%) were removed from the final statistical analysis because they did not perform the BeauBrain CST after the 16-week training program. In the control group, six participants (12.0%) were not included due to disease, withdrawal of consent, or loss of follow-up. Therefore, a total of 85 participants (84.1%) was included in the final analysis, including 41 participants (80.3%) in the intervention group and 44 participants (88.0%) in the control group. The average rate of smartphone application task-completion of the 41 intervention participants was 94.8%. The flow diagram for study participants is presented in Figure 1. There were no statistically significant differences between the intervention and control groups with regard to age, sex, years of education, or baseline score of K-MMSE or S-IADL (Table 3).

**Table 3:** Demographic characteristics of the study participants.

	Control group (*n* = 44)	Intervention group (*n* = 41)	*p* value
Age, years	70.75 ± 5.77	71.24 ± 6.02	0.700
Sex (M/F)	16/28	13/28	0.412
Education, years	13.66 ± 3.13	13.71 ± 2.69	0.940
K-MMSE	28.73 ± 1.66	28.90 ± 1.24	0.586
S-IADL	1.48 ± 1.61	1.27 ± 1.29	0.512

K-MMSE, Korean version of the Mini-mental State Examination; S-IADL, Seoul Instrumental Activities of Daily Living.

### 3.2 Intervention effects on cognitive performance

The group-by-time interaction effect was statistically significant in the language domain scores of the BeauBrain CST, suggesting that the intervention group showed a larger increase than the control group at 16 weeks (*p* = 0.019). There was no statistically significant interaction effect of the BeauBrain CST on the total score (*p* = 0.297), attention (*p* = 0.694), visuospatial (*p* = 0.081), memory (*p* = 0.331), or executive (*p* = 0.906) domain score.

To conduct further analysis of the language domain scores, where the group-by-time interaction effect was significant, the language domain subtest scores were analyzed. Statistically significant interaction effects were observed in the phonemic word fluency test (*p* < 0.001) but not in the semantic word fluency test (*p* = 0.677) and Difficult Naming Test (*p* = 0.295). The intervention group only showed a larger increase in the phonemic word fluency test at 16 weeks than did control group. The results of BeauBrain CST are presented in Table 4.

**Table 4:** Cognitive outcomes of the BeauBrain Cognitive Screening Test.

	Control group		Intervention group		*p* value		
	Pre	Post	Pre	Post	Group	Time	Group × Time
Total score	59.85 ± 10.59	62.19 ± 10.06	61.67 ± 8.60	65.26 ± 10.11	0.239	<0.001	0.297
Attention domain	8.27 ± 2.86	9.01 ± 2.36	8.68 ± 2.75	9.20 ± 2.38	0.537	0.035	0.694
Language domain	14.22 ± 3.33	14.68 ± 3.10	14.74 ± 2.94	16.42 ± 3.43	0.084	<0.001	0.019
Semantic fluency test	11.14 ± 2.54	11.84 ± 2.85	11.71 ± 2.99	12.15 ± 2.90	0.405	0.073	0.677
Phonemic fluency test	9.71 ± 3.92	8.93 ± 4.25	9.90 ± 3.35	12.95 ± 4.28	0.006	0.010	<0.001
Difficult Naming Test	10.63 ± 3.41	11.55 ± 2.97	11.00 ± 2.80	11.55 ± 2.97	0.780	<0.001	0.295
Visuospatial domain	7.48 ± 2.97	7.78 ± 2.88	7.52 ± 2.53	8.51 ± 2.29	0.487	0.002	0.081
Memory domain	20.70 ± 2.48	21.89 ± 2.66	21.13 ± 2.54	21.97 ± 2.81	0.633	<0.001	0.331
Executive domain	9.19 ± 2.59	8.83 ± 2.77	9.59 ± 2.33	9.17 ± 2.81	0.465	0.149	0.906

### 3.3 Intervention effects on frontal lobe activation

**Figure 2:**
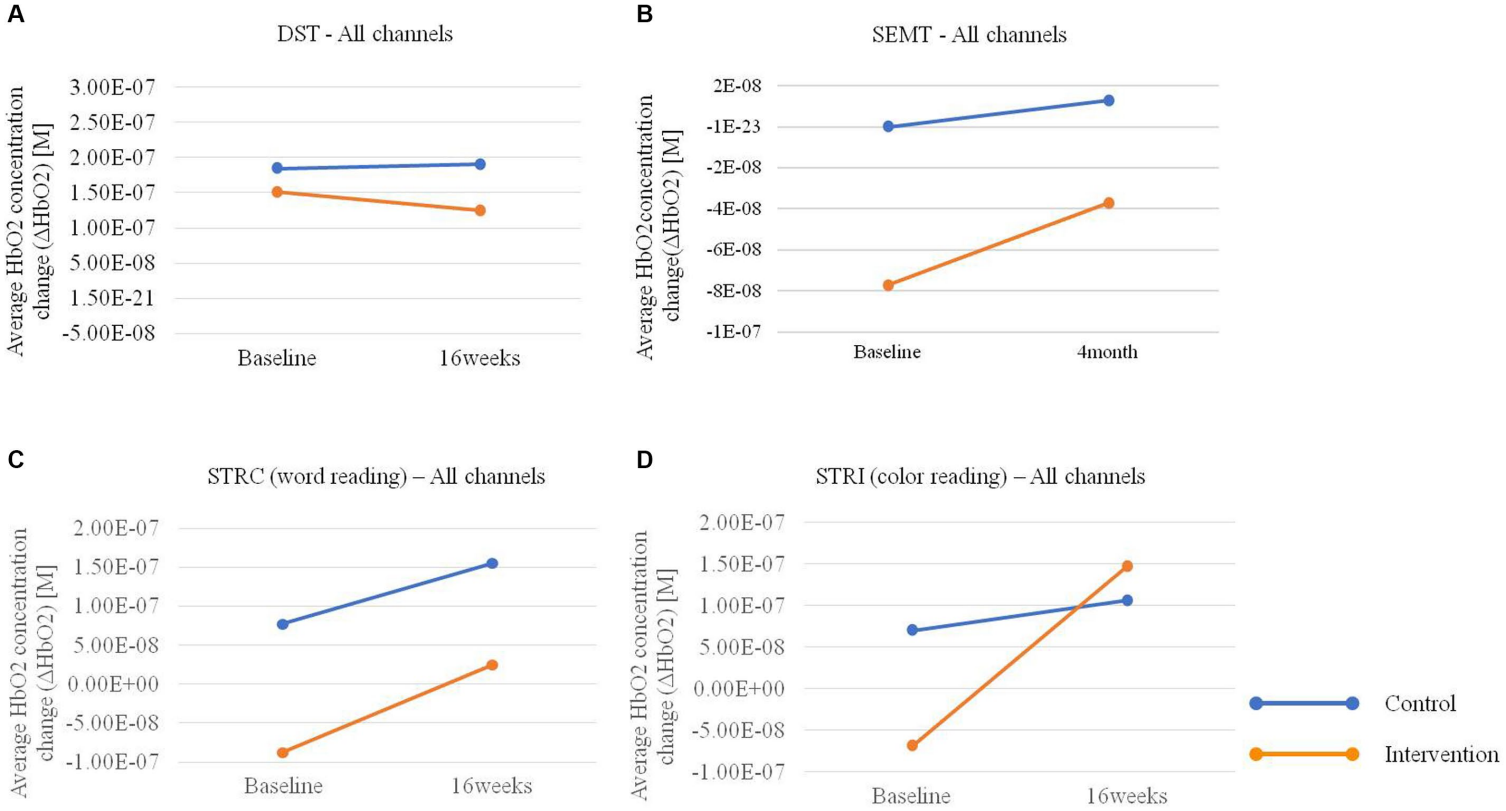
Average HbO2 concentration change (ΔHbO2) pre-post in the intervention and control groups for **(A)** Digit Span Test (DST), **(B)** Social Event Memory Test (SEMT), **(C)** Stroop Congruent (STRC) condition: word reading, and **(D)** Stroop Incongruent (STRI) condition: color reading tasks using fNIRS.

**Figure 3:**
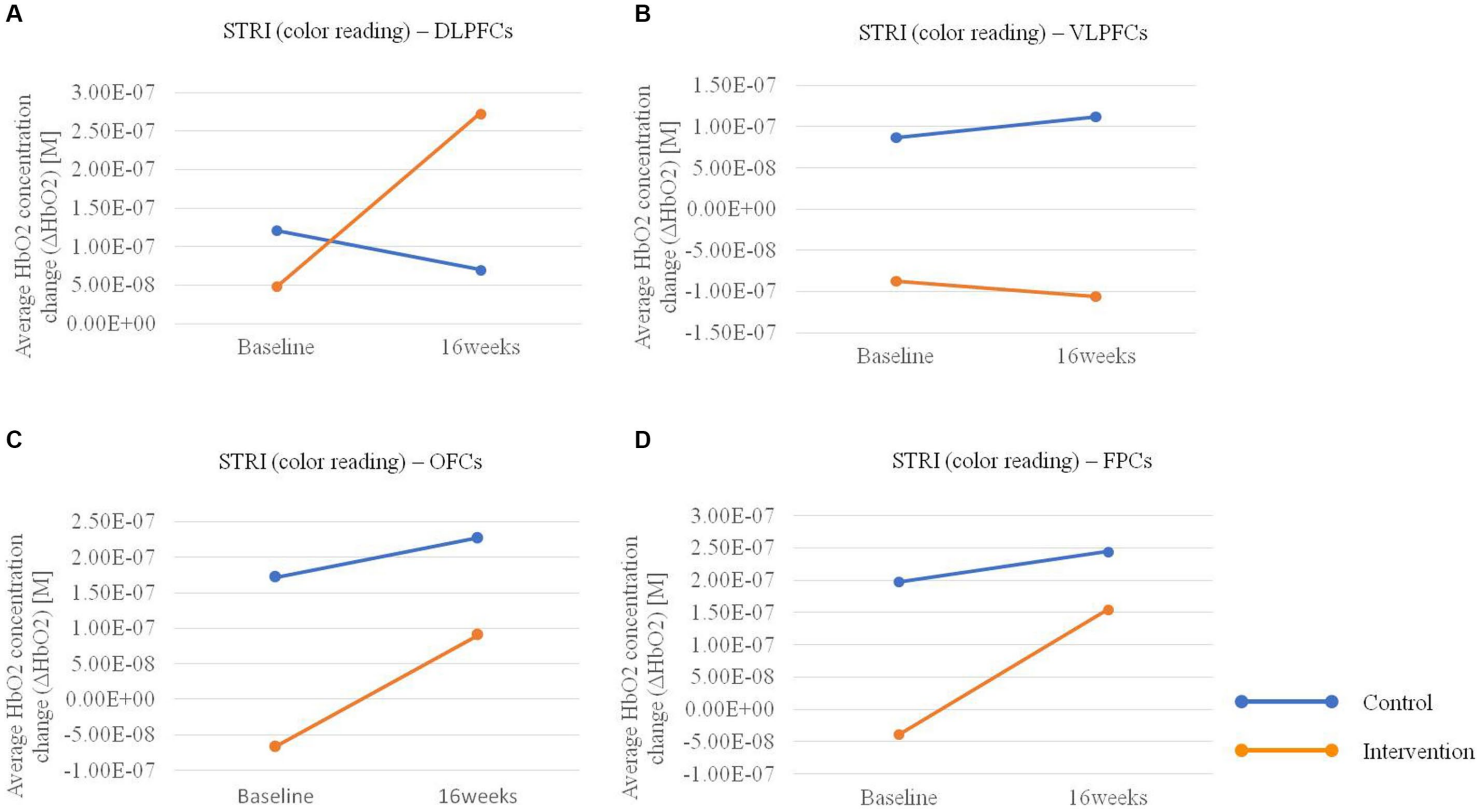
Average HbO2 concentration change pre-post in the intervention and control groups for the Stroop Incongruent (STRI) condition: color reading task in **(A)** bilateral dorsolateral prefrontal cortices (DLPFCs), **(B)** ventrolateral prefrontal cortices (VLPFCs), **(C)** orbitofrontal cortices (OFCs), and **(D)** frontopolar cortices (FPCs) regions.

**Figure 4:**
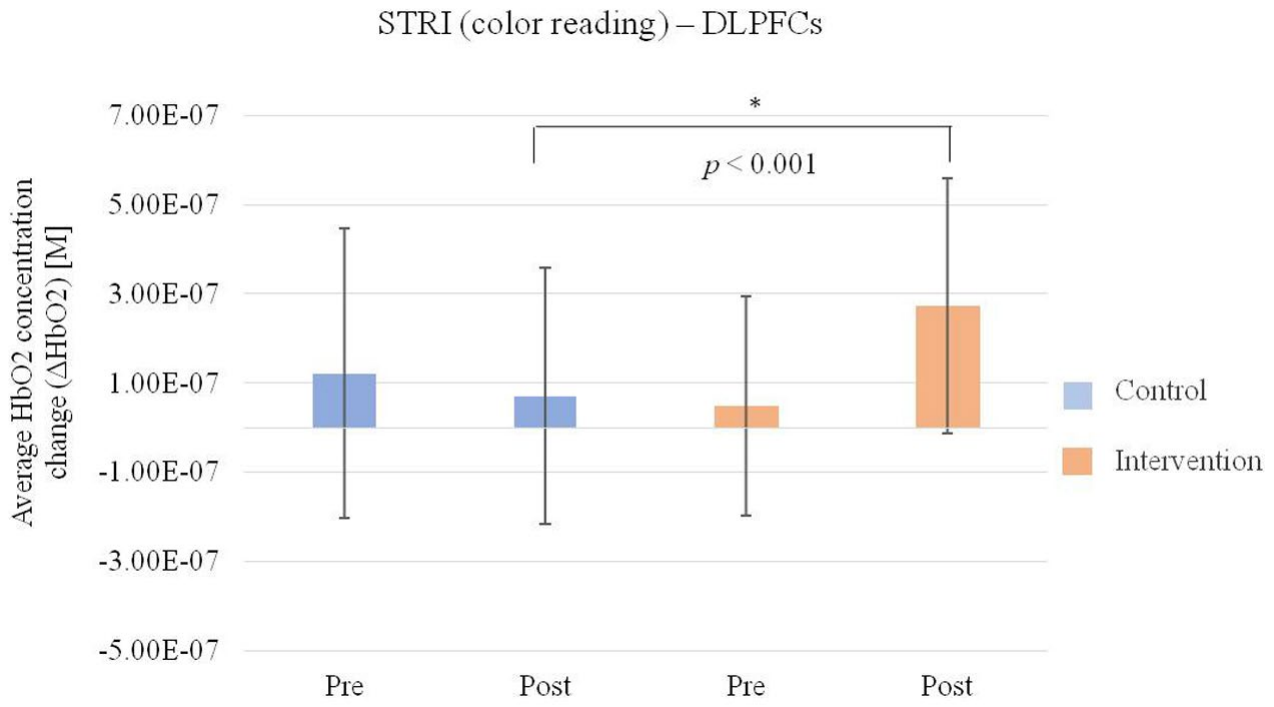
Average HbO2 concentration change (ΔHbO2) of bilateral dorsolateral prefrontal cortices (DLPFCs) regions for the Stroop Incongruent condition: color reading task pre and post intervention in the intervention and control group.

**Figure 5:**
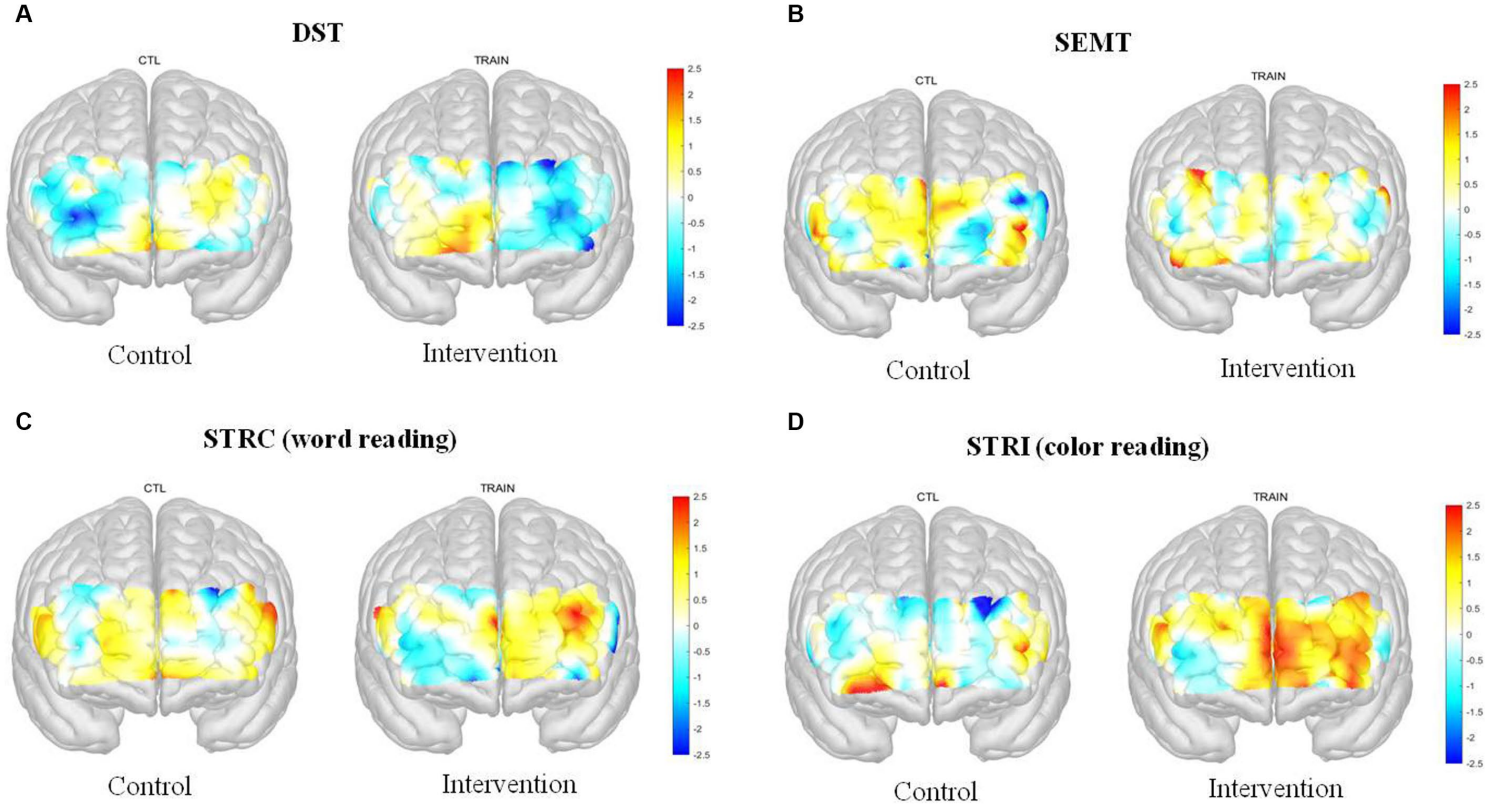
Post-Pre activation map of the fNIRS (*t*-value) for the Digit Span Test (DST), Social Event Memory Test (SEMT), Stroop Congruent (STRC): word reading, and Stroop Incongruent (STRI): color reading task in two groups.

In the hemodynamic response of the prefrontal cortex measured by all channels of fNIRS, the group-by-time interaction effect was only marginally statistically significant in the STROOP incongruent task (*p* = 0.055; Figure 2D). In the ROI analysis of this result, the statistically significant interaction effect was confirmed only in the DLPFCs (*p* < 0.001; Figures 3A, 4). After cognitive training, the intervention group exhibited a statistically significant increase in frontal lobe activation during the STROOP incongruent condition (color reading) task compared to the control group. But not in the VLPFCs (*p* = 0.879), OFCs (*p* = 0.371), FPCs (*p* = 0.274; Figures 3B–D). Furthermore, there was no significant interaction effect in the hemodynamic response of the prefrontal cortex on the DST, SEMT, and STROOP congruent tasks (Figures 2A–C). The post-pre activation maps of the two groups for each task are presented in Figure 5.

### 3.4 Intervention effects on other scales

When the secondary outcome measures were investigated, group-by-time interaction effects were not statistically significant for K-MMSE, K-ECog, B-ADL, SMCQ, GDepS, WHOQOL-BREF, SES, or AQS. These results are presented in Table 5.

**Table 5:** Secondary outcomes pre-and post-intervention.

	Control group		Intervention group		*p* value		
	Pre	Post	Pre	Post	Group	Time	Group × Time
K-ECog	1.65 ± 0.50	1.73 ± 0.58	1.68 ± 0.60	1.75 ± 0.57	0.797	0.047	0.916
B-ADL	1.98 ± 0.97	1.92 ± 0.90	2.09 ± 1.00	1.93 ± 1.05	0.752	0.192	0.567
SMCQ	4.43 ± 2.68	3.82 ± 2.78	5.10 ± 3.68	4.51 ± 3.27	0.292	0.006	0.946
GdepS	9.09 ± 7.05	8.68 ± 6.11	8.76 ± 6.08	8.88 ± 5.80	0.957	0.742	0.568
WHOQOL-BREF	71.43 ± 11.81	70.36 ± 11.01	72.35 ± 10.12	72.44 ± 9.21	0.491	0.501	0.450
SES	29.41 ± 4.59	28.93 ± 5.72	30.66 ± 3.90	30.57 ± 4.68	0.086	0.643	0.752
AQS	22.62 ± 4.80	21.73 ± 4.92	23.02 ± 4.84	22.32 ± 5.16	0.611	0.073	0.829

K-ECog, Korean Everyday Cognition; B-ADL, Bayer Activities of Daily Living; SMCQ, Subjective Memory Complaints Questionnaire; GDepS, Korean version of the Geriatric Depression Scale; WHOQOL-BREF, Korean version of the World Health Organization Quality of Life Scale (Abbreviated Version); SES, Self-efficacy Scale; AQS, Attention Questionnaire Scale.

## 4 Discussion

This study was performed to confirm the intervention effect of the BBT, an Android-based cognitive training software, on cognitive function and activation of the prefrontal cortex among older adults without dementia. The group-by-time interaction effect was statistically significant only in the language domain of the BeauBrain CST, only in the phonemic word fluency test (*p* < 0.001, the score increased from 9.90 to 12.95 in intervention group). Furthermore, in the hemodynamic response of the prefrontal cortex as measured by fNIRS, the group-by-time interaction effect was only statistically significant in the dorsolateral prefrontal cortex during the STROOP incongruent task (*p* < 0.001). Unexpectedly, there were no group-by-time interaction effects in the various scales for ADLs (i.e., K-ECog, B-ADL), depressive mood (i.e., GDepS), self-efficacy (i.e., SES), quality of life (i.e., WHOQOL-BREF), or subjective cognitive problems (i.e., SMCQ, AQS).

In terms of cognitive outcomes, the intervention group only showed a statistically significant improvement (after 16 weeks of cognitive training) in the phonemic word fluency test among the subtests of the language domain. Prior cognitive training studies have described improvements in language function, especially generative naming ability (Rojas et al., 2013; Mowszowski et al., 2014; Sherman et al., 2017). In the above paper, language function was mainly evaluated by the semantic & phonemic verbal fluency task and the Boston naming test. The summary effect size observed after cognitive training was moderate and significant. The treatment group showed significantly improved phonemic verbal fluency compared to that of the control group, which suggests that cognitive training facilitates left prefrontal function (Balietti et al., 2016; Barban et al., 2016). In addition, immediate memory, delayed memory, and language improved when cognitive training was performed for ≥6 months with a computerized brain exercise program, such as Brain Fitness (Dakim Inc., Santa Monica, CA, USA), in older adults (Miller et al., 2013; Shah et al., 2017). The Application-based Cognitive Training at Home (ACTH) intervention study also deployed the BBT cognitive training program in older adults without dementia in the community. After 1 year of daily cognitive training for 20–30 min, the ACTH intervention group showed significantly greater improvements in the total (increased from 60.4 to 68.8), memory (increased from 20.6 to 23.5), and language (increased from 15 to 17.4) domain scores of the BeauBrain CST compared to those of the control group (Jang et al., 2021). The ACTH study may have identified a larger number of cognitive domain effects than in our study because their intervention was longer than ours.

Unexpectedly, there was no statistically significant difference between the intervention and control groups with regard to ADLs, depressive mood, self-efficacy, quality of life, or subjective cognitive complaints. Although it is difficult to identify statistically significant improvements in psycho-behavioral factors after only 4 months of an intervention, our result differed from those of previous studies showing that cognitive training usually increases participants’ confidence and life satisfaction (Giuli et al., 2016; Abbadessa et al., 2022). However, our results seem to have arisen as a result of the psycho-social atmosphere during the study period, as psychological disturbances and anxiety levels among older adults populations increased with the COVID-19 pandemic.

The hemodynamic response of the prefrontal cortex (as measured by fNIRS) demonstrates the prefrontal cortical activation involved in cognitive tasks. Functional brain changes are expected to appear in brain areas that are related to cognitive skills that are being trained. Therefore, these brain activations provide valuable information about the mechanisms by which cognitive interventions improve cognitive function. In our study, the group-by-time interaction effect was only statistically significant in the dorsolateral prefrontal cortex during the STROOP incongruent task, which meant that the intervention group had greater prefrontal activation than did the control group during the task. Performance of the STROOP incongruent task involves the inhibitory control process that facilitates the suppression of automatic cognitive activity by new and non-automatic cognitive activity in cognitively conflicted conditions (Friedman and Robbins, 2022). Inhibitory control is one of the major functions of the prefrontal cortex, which can be strengthened with BBT training. Furthermore, our fNIRS result supported the better performance of the intervention group on the phonemic word fluency test of the BeauBrain CST, which is known to measure left prefrontal function.

Although few studies have reported cognitive training–induced changes in the frontal lobe, some have shown increased activation after cognitive training. Belleville et al. (2011) used fMRI to determine the effects of memory training on brain activation in older adults with MCI who were trained on memory encoding and retrieval strategies for 6 weeks (for 120 min each day). During the fMRI scan, participants were instructed to memorize a list of words (encoding) and to recognize previously learned words among new word lists. After training, brain activation increased in the frontal, temporal, and parietal areas (especially in the right inferior parietal lobe) of MCI patients. These results suggest that cognitive interventions induce changes in brain regions/networks that mediate processes (e.g., lateral frontoparietal cortex) and result in statistically significant neuronal changes that can be measured by brain imaging. The results also indicate that the brains of MCI patients remain highly plastic.

Balardin et al. (2015) examined differences in fMRI activation and deactivation patterns during episodic verbal memory encoding between individuals with MCI and healthy controls. Participants were scanned before and after a single session of strategic semantic training during the encoding of word lists. After training, both MCI and healthy controls exhibited increased activation in the frontoparietal network regions, including the left dorsolateral and ventrolateral prefrontal cortices. Only in patients with MCI had increased activation in the ventromedial prefrontal cortex and the right superior frontal gyrus related to semantic strategy implementation. In another study, Moon et al. (2022) evaluated the impact of a multi-domain lifestyle intervention, the SUPERBRAIN, on regional homogeneity (ReHo) in resting-state brain fMRI data. The group confirmed that the ReHo values in the left medial orbitofrontal gyrus and right superior parietal lobule were increased in the facility-based intervention group compared to those in the control group.

To enable the widespread use of the BBT program as a cognitive training tool, it should undergo validation to ensure quality and validity, and standardization based on various cognitive levels. Furthermore, ensuring the safety of the computerized assessment tool and strengthening its validity and reliability will be necessary. Future studies should use larger sample sizes, extend the follow-up period, and ensure the safety of computerized assessments to obtain more accurate data for multiple interpretations of the study results. Additionally, research encompassing diverse biomarker measurements will be required to support the evidence regarding the effects of cognitive training in older adults.

Our study has several limitations. First, when measuring the hemodynamic response of the prefrontal area using fNIRS, the performances of tasks carried out simultaneously (such as STROOP, SEMT, and DST) were not recorded. Therefore, the activation of DLPFCs in the intervention group was interpreted in the context of other cognitive outcomes. Second, as deoxyhemoglobin information is not provided during the analysis of hemodynamic data using fNIRS, there is limited information available to interpret the results. Third, our interpretations of the improvements in cognitive function and the underlying neural mechanisms are limited by the paucity of statistically significant findings. Fourth, we were unable to completely control other activities in the control group during the study period. Most participants in this study were outpatients who visited Samsung Medical Center. They paid attention to dementia prevention and health management even before their participation in this study. Thus, the outcome measure scores for some tasks in the control group showed a gradual improvement over time, introducing potential bias due to the control group’s engagement in various activities that could influence cognition. Fifth, we could not control the indirect psychological impacts of the COVID-19 pandemic on our participants during the study period. Therefore, it is presumed that results consistent with other studies related to psychological states after cognitive training were not obtained (Shah et al., 2017).

## Data availability statement

The original contributions presented in the study are included in the article/Supplementary material, further inquiries can be directed to the corresponding authors.

## Ethics statement

The studies involving humans were approved by the Institutional Review Board (IRB) of Samsung Medical Center (IRB no. 2019-06-083-006). The studies were conducted in accordance with the local legislation and institutional requirements. The participants provided their written informed consent to participate in this study. Written informed consent was obtained from the individual(s) for the publication of any potentially identifiable images or data included in this article.

## Author contributions

JinC, DN, HJ, and JuC: conceived and designed the experiments and writing – review and editing. JinC, EC, DA, and DK: performed the experiments. JinC, EC, DA, DK, IC, JihC, JoC, and JuC: data curation and formal analysis. JinC, EC, DA, and JuC: investigation. JinC, EC, DA, IC, and JihC: writing – original draft. All authors contributed to the article and approved the submitted version.

## Supplementary material

The Supplementary material for this article can be found online at:

https://www.frontiersin.org/articles/10.3389/fnagi.2023.1250420/full#supplementary-material
