# Supplementary material for: Efficacy of smartphone application-based multi-domain cognitive training in older adults without dementia
Source: Front Aging Neurosci. 2023 Nov 23;15:1250420. doi: 10.3389/fnagi.2023.1250420 (PMC10704020; doi:10.3389/fnagi.2023.1250420)
Supplement: Supplementary file 1 [file Presentation_1.pdf]

Supplementary Material

Randomization[1]

Consecutively recruited participants were randomly assigned to the intervention or control group using an electronic randomization tool (Sealed Envelope Ltd. 2019). A blocked randomization list was created. Randomization was stratified according to age (three age categories: ≥ 60 and < 70, ≥ 70 and < 80, and ≥ 80) and sex with the use of block sizes of 4, 6, and 8 in a 1:1 ratio. This randomization method was chosen to ensure balanced and unbiased group assignment, and it is expected to minimize potential confounding factors. Participants' demographic information, including age and sex, was considered in the randomization process to achieve representative groups. The use of different block sizes allows for flexibility and adaptability in the randomization process.

[Online] Available from: https://www.sealedenvelope.com/simple-randomiser/v1/lists.

References

1. Jang, H., et al., *Effects of smartphone application-based cognitive training at home on cognition in community-dwelling non-demented elderly individuals: a randomized controlled trial.* Alzheimer's & Dementia: Translational Research & Clinical Interventions, 2021. **7**(1): p. e12209.

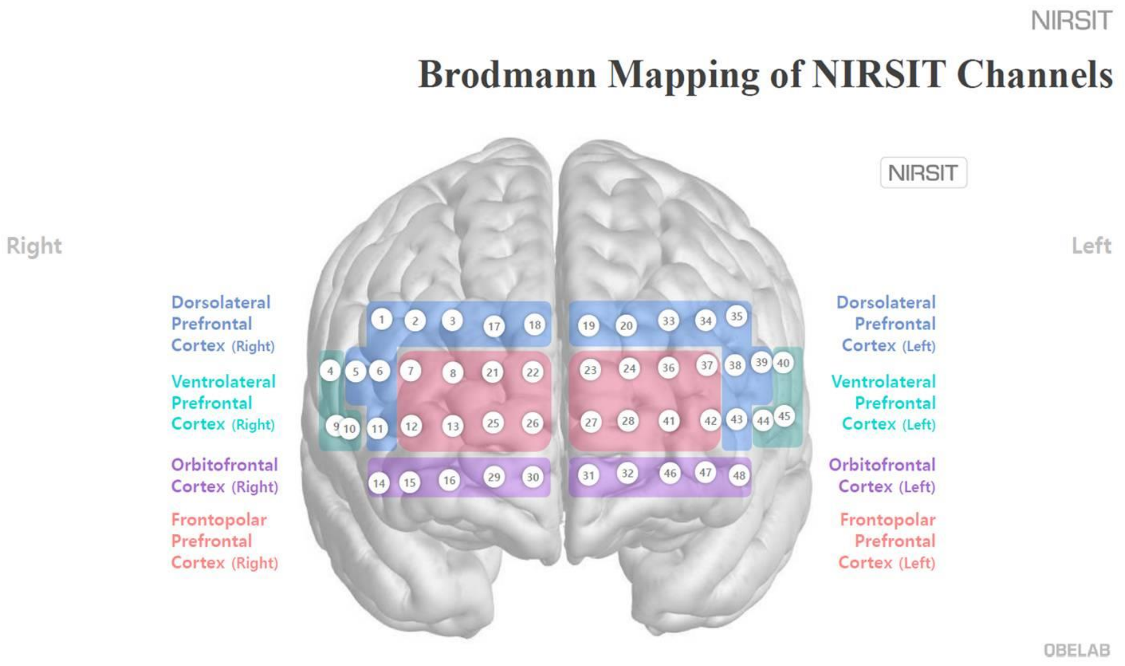

Fig. S1. fNIRS measurement configuration of 48 channels within the prefrontal cortex (reproduced from OBELAB. NIRSIT Channel Information, with permission of OBELAB Inc.34).

OBELAB, Inc., (2022). NIRSIT Channel Information, Seoul, Korea https://www.obelab.com/info/notice.php

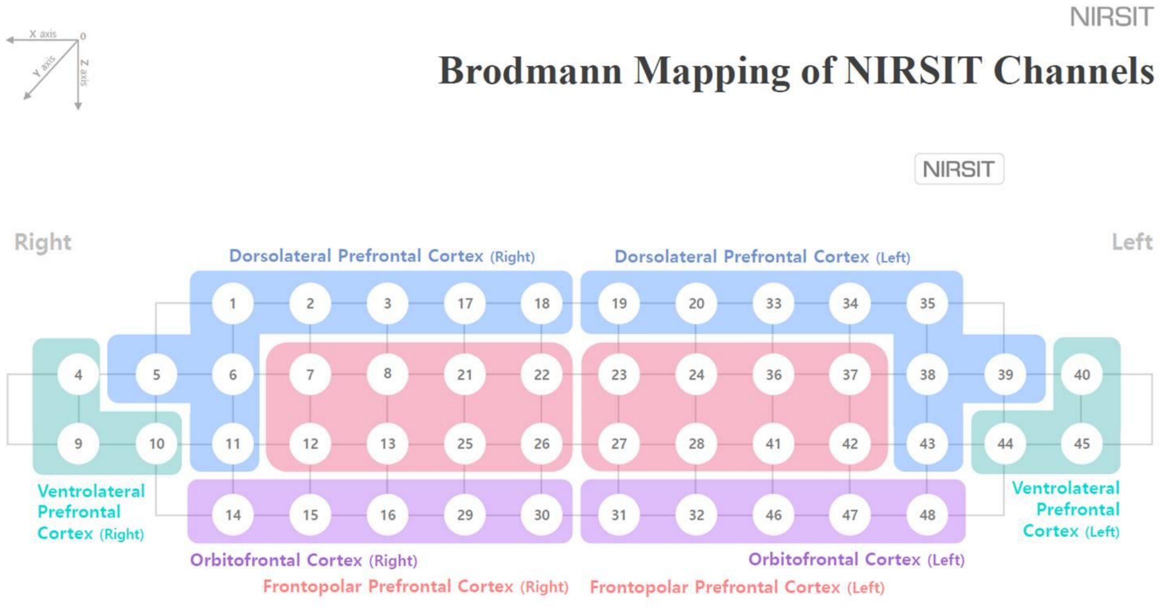

Fig. S2. fNIRS measurement configuration of 48 channels within the prefrontal cortex (reproduced from OBELAB. NIRSIT Channel Information, with permission of OBELAB Inc.34).

OBELAB, Inc., (2022). NIRSIT Channel Information, Seoul, Korea https://www.obelab.com/info/notice.php
